# Antenatal Risk Factors of Postpartum Depression at 20 Weeks Gestation in a Japanese Sample: Psychosocial Perspectives from a Cohort Study in Tokyo

**DOI:** 10.1371/journal.pone.0142410

**Published:** 2015-12-01

**Authors:** Yoshiyuki Tachibana, Tomoe Koizumi, Kenji Takehara, Naoko Kakee, Hiromi Tsujii, Rintaro Mori, Eisuke Inoue, Erika Ota, Keiko Yoshida, Keiko Kasai, Makiko Okuyama, Takahiko Kubo

**Affiliations:** 1 Division of Infant and Toddler Mental Health, Department of Psychosocial Medicine, National Center for Child Health and Development, Tokyo, Japan; 2 National Research Institute for Child Health and Development, Tokyo, Japan; 3 Department of Health Policy, National Research Institute for Child Health and Development, Tokyo, Japan; 4 Division of Bioethics, National Center for Child Health and Development, Tokyo, Japan; 5 Department of Psychosocial Medicine, National Center for Child Health and Development, Tokyo, Japan; 6 Department of Biostatistics, Clinical Research Center, National Center for Child Health and Development, Tokyo, Japan; 7 Japanese Midwives Association, Tokyo, Japan; 8 Department of Child Psychiatry, Kyushu University Hospital, Fukuoka, Japan; 9 Shirota Obstetrical and Gynecological Hospital, Tokyo, Japan; Chiba University Center for Forensic Mental Health, JAPAN

## Abstract

**Background:**

Prevalence of postnatal depression (PND) is high (Western countries, 10–15%; Japan, 17%). PND can cause parenting impairment and affect family health (e.g. child behaviors, cognitive development and physical health). This study aimed to reveal the risk factors of PND during the pregnancy period in a Japanese sample, and to identify the psychosocial risk factors of PND that should be appended to existing obstetric interview sheets. A cohort study with a Japanese sample was conducted.

**Methods:**

All 14 obstetrics hospitals in the Setagaya ward, Tokyo, Japan, participated in this study. Pregnant women who booked their delivery between December 2012 and May 2013 were enrolled. Data used for this study were collected at 20 weeks gestation, a few days and one month postnatal. The questionnaires consisted of psychosocial factors and the Edinburgh Postnatal Depression Scale (EPDS). To identify PND risk factors, multivariate analyses were performed.

**Results:**

A total of 1,775 women participated in this study. Eventually, the data of 1,133 women were used for the multivariate analyses. The demonstrated significant risk factors include EPDS score, primipara, “a perceived lack of family cohesion”, “current physical illness treatment” and “current psychiatric illness treatment”.

**Conclusion:**

This study highlights the importance of mental health screening using psychological measures during the pregnancy period. In addition, family environment, parity, physical and psychiatric illness should be paid attention by professionals in maternal and child health. The results also suggest that mothers’ feelings of developing their families should be supported.

## Introduction

The prevalence of postnatal depression (PND) is high. In Western countries, the prevalence of postnatal women experiencing a major depressive episode during this period is 10–15% [1]. In Japan, it is estimated to be at 17% [2]. PND can cause parenting impairment [3–5] and have negative effect on child behaviors [6], cognitive development [7] and physical health [8].

Obstetricians and midwives can serve as gatekeepers for detecting PND, as they see the women during pregnancy and the postnatal period. Attention to the risk factors of PND can lead to early detections and interventions for affected women. Previous studies performed in the U.S. and Europe revealed that depressed mood or anxiety during pregnancy, the level of social support, life events and psychiatric history, including previous experience of depression, poor marital relationship and low social status, are all important risk factors of depression in the postnatal period [1, 9, 10].

In Asia, two studies have suggested the sex of the newborn to be a risk factor of PND because of a societal preference for male offspring. In their studies, the other risk factors are consistent with previous studies; i.e. antenatal psychiatric morbidity, economic deprivation, low education, and marital disharmony and protective factors; education, support from extended family members, and employment [11, 12]. There are some studies that reported the antenatal risk factors for PND in Japan. In the study by Kitamura et al., in which 290 Japanese women expecting their first baby (among 1,159 women attending the antenatal clinic of the five university hospitals) were exmined, it was stated that PND is characterized by poor accommodation, dissatisfaction with sex of the newborn baby and with the emotional undermining [13]. Meanwhile, the Osaka Maternal and Child Health Study cohort study of 627 pregnant women conducted in Neyagawa city in Osaka revealed that job, especially for those holding a professional or technical full-time job, is significantly associated with a reduced risk of PND [14]. On the other hand, household income or maternal and paternal educational levels are not. Kokubu et al. reported that anxiety during pregnancy among 99 women attending four antenatal clinics predicts PND, and the effect of negative attitudes towards pregnancy on PND is possibly mediated by bonding failure [15]. Morikawa et al. revealed that social support and depressive symptoms among 877 women during pregnancy affect PND [16]. In view of these studies, we hypothesized that a large community-based cohort study that includes all the hospitals in one area conducted during the perinatal periods can add strong evidence of risk factors for PND. In addition, we think that perinatal professionals should pay attention to women at high risk for PND during the antenatal period. In Japan, home visits by public health nurses are conducted for almost all families with a newborn. The visits, done between one and four months after delivery, serve as an important aspect of the health policy in Japan to prevent child abuse and maltreatment, as well as to support child rearing and family mental health. At present, mental health screening during pregnancy is not a routine procedure in Japanese obstetric outpatient clinics. Although interview sheets about psychosocial aspects are commonly used, they are not considered to be reliable from the viewpoint of mental health practices. Psychosocial risk factors of PND in the pregnancy periods are useful for the screening at obstetric outpatient clinics to perform early interventions for women with high-risks of mental health.

The purpose of this study was to demonstrate the important aspects that should be paid attention to during the pregnancy period to predict PND. We conducted a cohort study on antenatal and postnatal mental health to determine the risk factors of PND in a Japanese sample. Using the cohort study data, we investigated the important psychosocial risk factors of PND.

## Methods

### Overall study design

**Fig 1:**
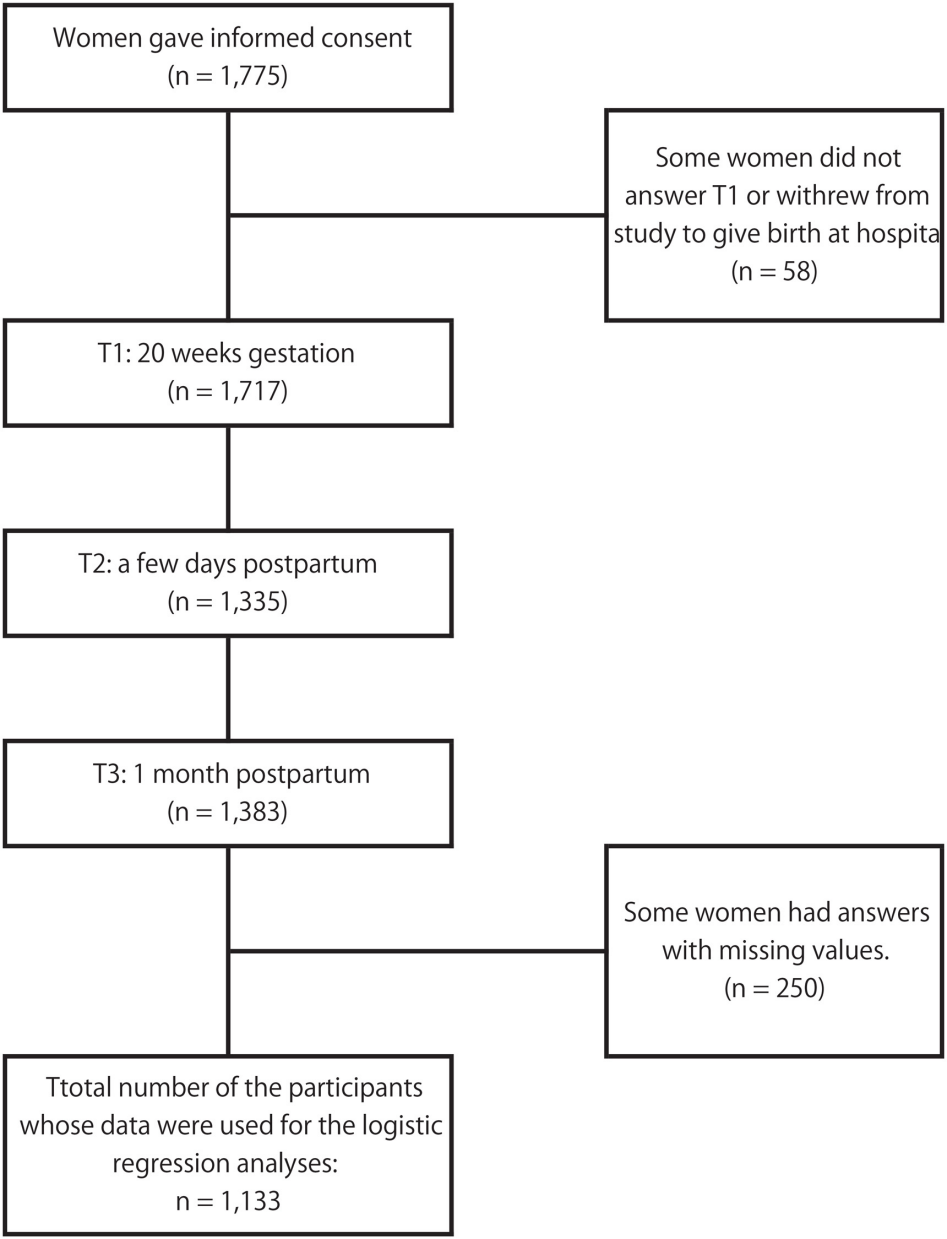
Flow chart of survey participation.

This was a cohort study. The project commenced in September 2012, with the cohort recruited from pregnant women in their second trimester in the Setagaya ward of Tokyo. Women who gave informed consent were asked to answer a survey at 20 weeks gestation and at five time points postnatal; the first few days, two weeks, one month, two months, and three months postnatal. At the first survey, subsequent questionnaires were distributed to the women unless they indicated their intention to withdraw from the study, or if they did not complete the questionnaire at the time point. Data for the survey at 20 weeks gestation, the first few days and one month after delivery were collected via the self-administered paper questionnaires or electronically using MOMONGA (Xware Corp, Tokyo, Japan), an iPad2 questionnaire application, at prenatal and postnatal check-ups, as well as during admission after delivery. One month postnatal questionnaires were distributed by care providers and returned to them directly at the hospital. For this study, we used the data at 20 weeks gestation (T1), a few days postnatal (T2), and one month postnatal (T3). Recruitment of the participants was conducted between December 2012 and May 2013. The flow of participants is shown in Fig 1.

### Participants

Participants are pregnant women who had been followed up at all the obstetrics outpatient clinics in the Setagaya ward (14 obstetrics hospitals), were under 20 weeks of gestation, and made appointment to deliver at those clinics. Exclusion criteria included women with intellectual disability, learning disorders or insufficient level of Japanese to complete the questionnaires, and those who did not deliver at the obstetrics hospitals in the Setagaya ward. In the event that a participant gave birth to a stillborn baby, the participant was withdrawn from the study. The research staff was updated with the situation by the perinatal staff. In turn, the research staff deleted the details of the participants from the delivery list for the self-administered paper questionnaires. Obstetricians, midwives and nurses supported the women in the same way as they would for all other cases. If a participant had severe mental health problems, medical care was administered by the perinatal staff. Subsequently, the participant was referred to another psychiatric clinic or hospital according to normal protocol.

### Recruitment

Two obstetric hospital networks in the Setagaya ward that were involved in the Japan Association of Obstetricians and Gynecologists were approached. Following presentations at regional network meetings, we met with the obstetric hospital directors who were interested to discuss the project details. A hospital would be accepted into the study if the obstetric doctors and nurses indicated support together with the director agreeing to the provision of necessary data. Eventually, all 14 obstetric hospitals in the Setagaya ward were accepted to be part of this cohort study. Participants were recruited via all the hospitals.

### Measures

The T1 questionnaire consisted of psychosocial questions and the Japanese version [17] of the Edinburgh Postnatal Depression Scale (EPDS) [18]. T1 was developed based on known predictors of PND and the standard obstetrical interview sheets in Japan. In addition, all the risk factors which were revealed by Robertson [10] and O’Hara [1] (mentioned in the introduction) were included. Low social status was defined by annual household income of less than two million yen. This income thresholds is a representative definition of the working poor in Japan [19]. Table 1 shows the demographic information collected at T1, which includes age, employment, education, income, plurality, parity, wanted pregnancy, psychiatric illness history, reproductive treatment history, type of pregnancy, delivery week.

**Table 1:** Basic characteristics of the participants.

		Mean (SD*)	Number	%	Missing value
Age (years)		35.06 (4.35)			19
Partner (+)					5
	Yes		1375	99.4	
	No		3	0.2	
Employment					0
	Full-time		563	40.7	
	Part-time		116	8.4	
	Temporary		42	3	
	Others (job (+))		85	6.1	
	Homemaker		577	41.7	
Educational level					7
	Graduate degree		105	7.6	
	University degree		723	52.3	
	Junior or technical college		389	28.1	
	High school		15	1.1	
	Junior high scool		11	0.8	
Auunal income					13
	<2 million yen		12	0.8	
	2–4.9 million yen		288	20.8	
	5–9.9 million yen		622	45	
	>10 million yen		448	32.4	
Plurality					2
	Singleton		31	2.2	
	Twin		1350	97.6	
	Triplet		0	0	
Parity					229
	Primiparous		621	44.9	
	Multiparous		533	38.5	
Wanted pregnancy					11
	Yes		1320	95.4	
	No		52	3.8	
Psychiatric illness history (+)					5
	Yes		17	1.2	
	No		1361	98.4	
Reproductive treatment history (+)					3
	Yes		74	5.4	
	No		1306	94.4	
Type of pregnancy					16
	Natural insemination		987	71.4	
	Guidance of preferable timing of fertilization		149	10.8	
	Artificial insemination		45	3.3	
	Extrauteral insemination		86	6.2	
	Microinsemination		98	7.1	
	Others		2	0.1	
Delivery week		38.96 (0.04)			0
Method of birth					227
	Spontaneous vaginal birth		935	67.6	
	Instrumental vaginal birth		123	8.9	
	Caesarean section		90	6.5	
	Others		8	0.6	

* indicates standard deviation.

The T2 data was about parity, and delivery week and method of birth. The T3 data was solely the EPDS.

### Data preparation

Data collected were entered into an electronic database. All manually processed questionnaires were double-checked for data quality. All measurements were examined for their ranges, distributions, means, standard deviations, outliers, and logical errors.

### Privacy protection

Databases needed for answering specific research questions were centrally built from databases concerning different time points of the study. All information enabling identification of the participants, with the exception the identification number of each participant, was erased from these databases.

### Statistical Analyses

The participants were classified as high-risk of PND or not using the cut-off score of the Japanese version of the EPDS (at 8/9) [17]. To explore the postnatal risk factors in preterm periods and soon after delivery, demographic, sociological, psychological, and psychiatric variables were included the preliminary bivariate analyses. Only “parity” among the T2 data was included in the analyses (“delivery week” and “method of birth” were not included in the analyses). All of the analyses were performed using the maximum samples with excluding missing values. We tested intergroup differences in categorical and continuous variables with the chi-square tests and Student’s t test for unpaired data, respectively. When a variable was significantly correlated to other similar independent variables), we selected variables for examining the determinants of PND risk factors. All *p* values reported were two-tailed. The variables which showed statistical significances were compared to the risk factors in the previous studies of Robertson and O’Hara [1, 10] to confirm if the variables covered were risk factors or not.

#### Analysis 1

To identify the risk factors of PND, variables with *p* value of less than 0.05 in the bivariate analysis were entered into a multivariate logistic regression model as independent variables and the classified two groups with the EPDS cut-off score as a dependent variable. The variables which showed *p* values of 0.05 or less were considered indicative of statistically significant risk factor of PND.

#### Analysis 2

To identify the significant psychosocial factors for interview sheets besides psychometric measurement of depression and anxiety, we performed a logistic regression analysis using the same variables besides the EPDS for independent variables and the classified two groups with the EPDS cut-off score as a dependent variable. All data analyses were performed with SPSS version 21.0 J for Windows (SPSS Inc., Tokyo, Japan).

Multicollinearities of the logistic regression models of Analyses 1 and 2 were estimated. They were performed in the coefficiency estimates of the linear regression in which the same dependent variable and independent variables of the logistic regression analysis were used. We judged the presence of multicollinearity if tolerance values were less than 0.4 and the variance inflation factor (VIF) was greater than 2.5 using Allison’s criteria [20].

### Subanalysis

The antenatal risk factor of PND may also have a strong relationship with antenatal depression. Since antenatal depression was measured by EPDS, we performed subanalyses classifying the participants into two groups: women at high risk of depression during pregnancy and women who were not at high risk using EPDS cut-off score 9 at T1 (Analysis 3 and Analysis 4, respectively). We performed the same logistic regression analyses for the two groups, respectively.

## Results

The flow of the number of the participants is shown in Fig 1. A total of 1,775 women gave informed consent. Of them, 1,717 completed the T1 questionnaire and 1,383 completed the T2 questionnaire. Table 1 shows the demographic characteristics of the sample. The data of the women who completed the T2 were used for the bivariate analyses (some data with missing values were excluded in each analysis). Since 250 of them had missing values, the data of 1,133 women were used for the multivariate analyses.

The results of the bivariate analysis are shown in Table 2. Finally, the factors which showed statistical significances (p <0.05) in the bivariate analyses were input into the logistic regression analyses. These factors included the important factors which Robertson and O’Hara mentioned in the previous studies [1, 10].

**Table 2:** The results of the bivariate tests of the factors at 20 weeks gestation with the mental health risk in the Edinburgh Postpartum Depression Scale at one month postpartum).

		Total number	At High risk**		Not at high risk**		
			Number	%	Number	%	p value
EPDS at 20 weeks gestation		1327					0.00***
	At high risk*		53	41.4	75	58.6	
	Not at high risk*		116	9.7	1083	90.3	
Young age pregnancy (less than 20 years old)		1364					0.70
			0	0.0	1	100.0	
			177	13.0	1186	87.0	
Elder age pregnancy (more than 34 years old)		1364					0.16
	Yes		108	14.1	658	85.9	
	No		69	11.5	529	88.5	
Multiple pregnancy		1381					0.11
	Yes		7	22.6	24	77.4	
	No		174	12.9	1176	87.1	
Having a job		1383					0.87
	Yes		108	13.4	699	86.6	
	No		73	12.7	502	87.3	
Job type (if any)		1383					0.32
	None		74	12.8	503	87.2	
	Full-time		69	12.3	494	87.7	
	Part time		28	17.7	130	82.3	
	Freelance/Others		10	11.8	75	88.2	
Weekly working hour		1383					0.55
	No job		74	12.8	505	87.2	
	Less than 15hours		16	17.6	75	82.4	
	15~29hours		8	10.0	72	90.0	
	30~34hours		9	11.8	67	88.2	
	35~39hours		14	11.1	112	88.9	
	40~48hours		41	16.1	214	83.9	
	49~59hours		10	8.9	102	91.1	
	More than 60hours		4	12.1	29	87.9	
	Not fixed		5	16.1	26	83.9	
Having Overtime works after 10 PM		1383					0.46
	No job		74	12.9	501	87.1	
			11	9.6	103	90.4	
			96	13.8	598	86.2	
Having a partner		1378					0.30
	Yes		180	13.1	1195	86.9	
	No		1	33.3	2	66.7	
Emotional supports by partner		1379					0.09
	Very much		103	11.8	768	88.2	
	Much		71	14.9	405	85.1	
	Slightly, some of the time		5	18.5	22	81.5	
	Not at all		2	40.0	3	60.0	
Practical support by partner		1374					0.20
	Very much		66	11.4	513	88.6	
	Much		97	15.2	543	84.8	
	Slightly, some of the time		14	10.8	116	89.2	
	Not at all		4	16.0	21	84.0	
Emotional supports by others besides partner		1379					0.00***
	Very much		159	12.3	1135	87.7	
	Not so much		21	24.7	64	75.3	
Practical support by others besides partner		1379					0.01***
	Very much		135	11.9	999	88.1	
	Not so much		45	18.4	200	81.6	
A perceived lack of family cohesion		1380					0.00***
	Very much		67	9.2	665	90.8	
	Much		101	17.3	484	82.7	
	Slightly, some of the time		13	21.0	49	79.0	
	Not at all		0	0.0	1	100.0	
Parity		1154					0.00***
	Primipara		31	5.8	502	94.2	
	Pruripara		109	17.6	512	82.4	
Feeling to have been abused when a child		1378					0.02***
	Yes		9	25.0	27	75.0	
	No		159	12.5	1117	87.5	
	Not sure		13	19.7	53	80.3	
Feeing to have been received love and care in growing up		1381					0.00***
	Yes		160	12.2	1148	87.8	
	No		4	33.3	8	66.7	
	Not sure		17	27.9	44	72.1	
Current physical illness treatment		1380					0.00***
	Yes		31	21.8	111	78.2	
	No		150	12.1	1088	87.9	
Current psychiatric illness treatment		1381					0.00***
	Yes		9	45.0	11	55.0	
	No		172	12.6	1189	87.4	
Past psychiatric treatment history		1378					0.00***
	Yes		43	23.2	142	76.8	
	No		136	11.4	1057	88.6	
Infertility treatment history		1380					0.80
	Yes		9	12.2	65	87.8	
	No		172	13.2	1134	86.8	
Pregnant type		1367					0.67
	Natural conception		121	12.3	866	87.7	
	Guidance on timing method		25	16.8	124	83.2	
	Artificial insemination		7	15.6	38	84.4	
	In-vitro fertilization		10	11.6	76	88.4	
	Micro fertilization		14	14.3	84	85.7	
	Others		0	0.0	2	100.0	
Unwanted pregnancy		1372					0.11
	Yes		176	13.3	1144	86.7	
	No		3	5.8	49	94.2	
How did you feel when you found that you were pregnant this time?		1377					0.16
	I was happy.		120	12.0	880	88.0	
	It was not expected, but I was happy.		40	14.4	237	85.6	
	It was not expected, so I didn't know what to do.		15	20.5	58	79.5	
	I was at a loss.		2	28.6	5	71.4	
	I didn't feel anything special.		3	15.0	17	85.0	
Stressful life events within a year		1381					
	Moving						0.04***
	-Yes		59	16.2	305	83.8	
	-No		122	12.0	895	88.0	
	Started to live together with parents and/or parents-in-law						0.61
	-Yes		5	16.1	26	83.9	
	-No		176	13.0	1174	87.0	
	Separation between husband and wife						0.08
	-Yes		0	0.0	20	100.0	
	-No		181	13.3	1180	86.7	
	Divorce						0.03***
	-Yes		2	50.0	2	50.0	
	-No		179	13.0	1198	87.0	
	Death in family						0.34
	-Yes		14	16.5	71	83.5	
	-No		167	12.9	1129	87.1	
	Major illness and/or injury						0.33
	-Yes		7	18.4	31	81.6	
	-No		174	13.0	1169	87.0	
	Taking care of a sick or aged family member						0.73
	-Yes		10	14.5	59	85.5	
	-No		171	13.0	1141	87.0	
	Leaving or losing one's job						0.00***
	-Yes		32	22.2	112	77.8	
	-No		149	12.0	1088	88.0	
Annual household income		1370					0.12
	On welfare		0	0.0	2	100.0	
	<2 million yen		3	30.0	7	70.0	
	2–4.9 million yen		39	13.5	249	86.5	
	5–9.9 million yen		91	14.6	531	85.4	
	>10 million yen		46	10.3	402	89.7	
Educational qualification		1376					0.35
	Junior high school diploma		2	18.2	9	81.8	
	High school diploma		26	19.5	107	80.5	
	Specialized vocational college		23	13.9	142	86.1	
	Junior college		27	12.1	197	87.9	
	Specialized vocational high school		1	6.7	14	93.3	
	Four year college/university		87	12.0	636	88.0	
	Graduate school		14	13.3	91	86.7	
Frequency of drinking		1382					0.25
	None		177	13.5	1133	86.5	
	1 day~2 days/week		3	6.7	42	93.3	
	3~4 days/week		1	5.6	17	94.4	
	5~7days/week		0	0.0	9	100.0	
Amount of drinking at one time		1383					0.60
	None		176	13.4	1142	86.6	
	1. Less than a bottle(500ml) of beer, or 180ml of sake, two glasses of wine		4	7.8	47	92.2	
	2. More than 3 bottles of beer		0	0.0	2	100.0	
	Amount between 1 and 2.		1	8.3	11	91.7	
Amount of smoking a day		1377					0.46
	None		175	12.9	1184	87.1	
	Less than 5 cigarettes		1	10.0	9	90.0	
	Between 5~9 cigarettes		2	33.3	4	66.7	
	Between 10~19 cigarettes		0	0.0	2	100.0	
	Twenty and above		0	0.0	0	0.0	
Frequency of gambling in a month		1367					0.03***
	None		176	12.9	1186	87.1	
	Once or twice		1	25.0	3	75.0	
	3~4times		1	100.0	0	0.0	
	5times and above		0	0.0	0	0.0	
Low social status (Less than 2 million yen annual income)		1370					0.22
	Yes		3	25.0	9	75.0	
	No		176	13.0	1182	87.0	
Two or more stressful events		1381					0.00***
	Yes		32	21.2	119	78.8	
	No		149	12.1	1081	87.9	

*, **: The participants was divided into two groups, i.e. "At high risk" or "Not at high risk" of postpartum depression using the cut-off score of the Japanese version of the Edinburgh Postpartum Depression Scale (8/9) at 20 weeks' gestation and at 1 month after delivery, respectively. The scores of the "At high risk" group were not lower than 8. The scores of the "Not at high risk" group were not greater than 9. "p value" means the value of the Chi-square test for each variable between the two groups. "Total number" means the number of the participants which were in the analyses.

*** indicates statistically significant (p < 0.05) in the analyses.

The results of Analysis 1 are shown in Table 3. The EPDS score (p value (p) <0.01, Odds ratio (OR) = 5.45 [95% confidential interval of the odds ratio (95%CI)] = 3.22–9.22) and primipara (p <0.01, OR = 3.38[95% CI = 2.09–5.45]) were the statistically significant risk factors of PND.

**Table 3:** The results of the multivariate analysis of predictive factors at 20 weeks gestation for postpartum depression.

Predictors	p value	AOR (95% CI)
High risk with the EPDS at 20 weeks gestation	<0.01*	5.45 (3.22–9.22)
Emotional support by others besides partner	0.21	1.62 (0.77–3.40)
Practical support by others besides partner	0.29	1.32 (0.79–2.21)
A perceived lack of family cohesion	0.17	1.26 (0.91–1.76)
Primipara	<0.01*	3.38 (2.09–5.45)
Feeling to have been abused when a child	0.47	1.20 (0.73–1.98)
Feeling to have been received love and care in growing up	0.41	1.20 (0.78–1.84)
Current physical illness treatment	0.08	1.76 (0.93–3.32)
Current psychiatric illness treatment	0.13	3.22 (0.71–14.67)
Past psychiatric or psychological history	0.56	1.18 (0.67–2.07)
Moving	0.92	1.02 (0.66–1.59)
Divorce	0.58	2.37 (0.12–48.05)
Leaving or losing one's job	0.22	1.42 (0.82–2.46)
Frequency of gambling in a month	0.08	3.96 (0.84–18.75)

AOR, 95% CI and p value indicates the values of the Adjsusted Odds ratios and 95% confidence intervals of the odd ratios and p values in the logistic regression analysis, respectively.

* indicates statistical significance in the analysis (p < 0.05).

The results of Analysis 2 are shown in Table 4. Significant risk factors include “a perceived lack of family cohesion” (p <0.01, OR = 1.58[95% CI = 1.16–2.15]), primipara (p < 0.01, OR = 3.06[95% CI = 1.94–4.81]), “current physical illness treatment” (p = 0.02, OR = 2.02[95% CI = 1.12–3.64]), and “current psychiatric illness treatment” (p = 0.03, OR = 3.94[95% CI = 1.01–15.39]).

**Table 4:** The results of the of multivariate analysis of predictive factors at 20 weeks gestation for postpartum depression except for the Edinburgh Postpartum Depression Scale.

Predictors	p value	AOR (95% CI)
Emotional support by others besides partner	0.27	1.48 (0.74–2.96)
Practical support by others besides partner	0.17	1.40 (0.87–2.26)
A perceived lack of family cohesion	<0.01*	1.58 (1.16–2.15)
Primipara	<0.01*	3.06 (1.94–4.81)
Feeling to have been abused when a child	0.37	1.23 (0.78–1.92)
Feeling to have been received love and care in growing up	0.15	1.33 (0.90–1.95)
Current physical illness treatment	0.02*	2.02 (1.12–3.64)
Current psychiatric illness treatment	0.04*	3.94 (1.01–15.39)
Past psychiatric or psychological history	0.08	1.58 (0.95–2.62)
Moving	0.26	1.27 (0.84–1.91)
Divorce	0.37	3.37 (0.24–46.89)
Leaving or losing one's job	0.09	1.55 (0.93–2.57)
Frequency of gambling in a month	0.17	2.99 (0.63–14.25)

AOR, 95% CI and p value indicates the values of the Adjsusted Odds ratios and 95% confidence intervals of the odd ratios and p values in the logistic regression analysis, respectively.

* indicates statistical significance in the analysis (p < 0.05).

The results of coefficiency statistics for multicollinearity are shown in Table 5. The estimation of the tolerance value and VIF in the models was less than 0.4 and 2.5, respectively, which revealed that there was no multicollinearity in Analysis 1 and Analysis 2 models.

**Table 5:** The results of coefficiency statistics of predictive factors used for the multivariate analysis at 20 weeks gestation for postpartum depression.

Predictors	Tolerance	VIF
High risk with the EPDS at 20 weeks gestation	0.90	1.11
Emotional support by others besides partner	0.88	1.13
Practical support by others besides partner	0.87	1.15
A perceived lack of family cohesion	0.93	1.08
Primipara	0.92	1.08
Feeling to have been abused when a child	0.88	1.14
Feeling to have been received love and care in growing up	0.88	1.14
Current physical illness treatment	0.91	1.10
Current psychiatric illness treatment	0.85	1.18
Past psychiatric or psychological history	0.88	1.13
Moving	0.95	1.06
Divorce	0.99	1.01
Leaving or losing one's job	0.94	1.07
Frequency of gambling in a month	0.98	1.02

Coefficiency statistics means the results of the multicollinearity test in the linear regression in which the same dependent variable and independent variables of the logistic regression analysis were used. Tolerance and VIF means tolerance value and variance inflation factor in the multicollinearity test, respectively.

The results of the subanalyses (Analyses 3 and 4) are shown in Table 6. “Divorce” was excluded in Analyses 3 and 4 because of low frequency with the one independent variable. “Frequency of gambling in a month” was also excluded in Analysis 3 because there were no participants who took up gambling in those at high risk of depression at T1. There was no statistical significant predictor, but “leaving or losing one’s job” was a marginal significant predictor in Analysis 3. In analysis 4, “a perceived lack of family cohesion” (AOR 1.55, 95% CI 1.05–2.29, p value 0.03) and “primipara” (AOR 5.52, 95% CI 2.94–10.38, p value<0.01) were shown as significant predictors of PND.

**Table 6:** Odds comparisons of woment at high risk and not at high risk of depression during pregnancy.

	Analysis 3		Analysis 4	
	At high risk		Not at high risk	
Predictors	AOR (95% CI)	p value	AOR (95% CI)	p value
Emotional support by others besides partner	1.41 (0.31–6.35)	0.66	1.81 (0.77–4.24)	0.17
Practical support by others besides partner	1.51 (0.51–4.51)	0.46	1.24 (0.68–2.28)	0.48
A perceived lack of family cohesion	0.83 (0.43–1.58)	0.57	1.55 (1.05–2.29)	0.03*
Primipara	1.02 (0.39–2.64)	0.97	5.52 (2.94–10.38)	<0.01*
Feeling to have been abused when a child	1.21 (0.47–3.11)	0.69	1.13 (0.62–2.06)	0.68
Feeling to have been received love and care in growing up	1.10 (0.51–2.34)	0.81	1.25 (0.74–2.09)	0.40
Current physical illness treatment	2.36 (0.70–7.98)	0.17	1.54 (0.70–3.36)	0.28
Current psychiatric illness treatment	1.32 (0.15–11.38)	0.80	7.04 (0.94–52.57)	0.06
Past psychiatric or psychological history	1.45 (0.52–4.09)	0.48	1.19 (0.61–2.35)	0.61
Moving	0.60 (0.24–1.50)	0.28	1.20 (0.73–1.97)	0.48
Divorce	Excluded			
Leaving or losing one's job	3.26 (0.99–10.67)	0.05	1.15 (0.60–2.20)	0.67
Frequency of gambling in a month	N/A		3.90 (0.81–18.84)	0.09

AOR, 95% CI and p value indicates the values of the Adjsusted Odds ratios and 95% confidence intervals of the odd ratios and p values in the logistic regression analysis, respectively.

* indicates statistical significance in the analysis (p < 0.05).

"Excluded" indicates that the variable was excluded in the logistic regression analyses because of low frequency. N/A indicates that the there was no participants and the variable was not included in the analysis.

## Discussion

Our study has two central findings. First, EPDS can be used as a strong mental health screening tool at 20 weeks gestation to predict PND. Second, there are four important psychosocial risk factors of PND: i.e. a perceived lack of family cohesion, primipara, current physical illness treatment history, and current psychiatric illness treatment history. It is a new finding that a perceived lack of family cohesion is an important psychosocial risk factor of PND. Interestingly, we also found that family cohesion is a more important factor for predicting PND compared to partner support. The other findings are consistent with previous reports.

We show that EPDS is a strong predictor of PND, further supporting the usefulness of antenatal mental health screening. EPDS has been used in the antenatal setting as the Edinburg Depression Scale (EDS) [21, 22]. In Japan, it is not common to use EDS for mental health screening. We propose its use in mental health screening so as to facilitate prompt antenatal interventions in women who are symptomatic or “at risk” for PND.

When considering only the psychosocial aspects, factors such as “a perceived lack of family cohesion”, primipara, “current physical illness treatment”, and “current psychiatric illness treatment” were revealed to be very important to predict PND, according to the interview sheets at obstetric outpatient clinics during the pregnancy period. In terms of the strongest risk factors of PND during pregnancy, Robertson et al. demonstrated factors such as depression, anxiety, stressful life events, low level of social support, and previous history of depression risk factor [10], while O’Hara showed past history of psychopathology and psychological disturbance, poor marital relationship and low social support, and stressful life events. In addition, a low social status showed a small but significant predictive relation to PND [1]. The results of our chi-square analyses showed that all these factors are related to PND in Japanese samples.

Both Analyses 1 and 2 revealed the importance of primipara as a risk factor of PND. The results are consistent with previous studies [23–25]. This is likely due to the fact that women who are pregnant for the first time experience greater psychological stresses compared to those have had gone through childbirth. Furthermore, the tasks and responsibilities of childcare are new to primipara women, and may add to the stress level.

A perceived lack of family cohesion on the women’s part is a new risk factor of PND that has never been reported before. In view of this, professionals in maternal and child health should be more attentive and supportive towards the mother’s feelings for her family during pregnancy. The item “a perceived lack of family cohesion” was added to our questionnaire because we wanted to investigate the importance of the women’s personal relationships when it comes to predicting PND. Family cohesion is defined as shared affection, support, helpfulness, and caring among family members [26–28].

Many studies have demonstrated the importance of emotional and practical support for pregnant mental health [10]. Morikawa et al. [16] showed that a larger number of supportive persons during pregnancy helps protect against postpartum depression, but that satisfaction rating with those supports does not. They used the Japanese version of Social Support Questionnaire 6 [29] (J-SSQ) which had been standardized by Furukawa et al [30]. The questionnaire used in this study did not query participants on the number of supportive persons nor the level of satisfaction with the support. In this study, we classified social support for pregnant women into four categories: emotional support by the partner, emotional support by others besides the partner, practical support by the partner, and practical support by others besides the partner. J-SSQ’s six items can be considered to be related to either practical supports or emotional support, or both. We found that “a perceived lack of family cohesion” was a more important risk factor than emotional and practical support by the partner. This result reflects the unique characteristics of the Japanese society, whereby working hours are generally longer than other countries [31] and many Japanese men find it difficult to have sufficient time to support their partners. While this may typically affect the mental status of mothers, we found that this is not the case in Japan. Japanese women are known for their sense of self-sacrificial love for their family [32]. Therefore, even when there is a lack of support from their partners, they could still feel happy as long as there is a satisfactory level of family cohesion.

We found that mothers who were under physical illness treatment were likely to have greater mental stress, together with the possibility of having psychosomatic symptoms. Perinatal staffs are usually the ones who check for physical problems in pregnant women. Our results suggest that perinatal stuff should also pay attention to the risk factors of postpartum depression. Physical illness treatment has not been addressed as a risk factor of PND in previous studies [1, 9, 10, 33]. Our unique finding may be due to the characteristics of the public health services in Japan. All Japanese citizens are enrolled in a health insurance system which allows them to receive medical services at reasonable prices (70% of medical fees are subsidized). General hospitals, where obstetricians can seamlessly refer pregnant women with physical illnesses to other department doctors, are also easily accessible. However, further studies are still needed to investigate why this factor is significant in Japan. Some mediating factor (i.e. psychosomatic aspects and social and cultural aspects including public health and medical services) may affect this factor.

From our multivariate analysis, current psychiatric illness treatment was identified as an important risk factor of PND, whereas past psychiatric history was not risk factor. However, there is a large number of literature which suggested that both are important risk factors for PND [34, 35]. This additional information can be used by midwives and obstetricians when tending to pregnant women with psychiatric problems.

The results of the subanalyses suggest that “primipara” and “a perceived lack of family cohesion” are important risk factors for those who did not have antenatal depression. In order words, perinatal professionals for postnatal mental care should pay close attention to these factors regardless of whether the pregnant individual had depression or not. There was a discrepancy of AOR between those who were “at high risk” and those who were “not at high risk” of depression during pregnancy in “a perceived lack of family cohesion”. The results suggested that for those without depression, a perceived lack of family cohesion can be a more severe psychological burden compared to depression. On the other hand, the results also suggested that a perceived lack of family cohesion may sometimes be a protective factor against PND. Those who have depression during pregnancy often have family relationship problems (e.g. low level of intimacy with the husband) [36]. The results suggest that, when depression during pregnancy was coupled with family problems, the pregnant woman may learn to be objective rather than being confronted and anguished by the problems—which could be a protective factor against worsening depression. The results of AOR of those who were “not at high risk” of antenatal depression being much larger than that of those who were “at high risk” suggest that primipara is a major antenatal risk factor for PND, especially for those who were not depressive during pregnancy. The results of AOR gap between “at high risk” and “not at high risk” suggest that, if antenatal depression existed, the condition of women with physical illness may worsen compared to those “without depression”. Collaborative care that integrates mental and physical care [37, 38] for those who have physical illness during pregnancy may be important in preventing PND. The results of AOR of “current psychiatric history” of those who were “not at high risk” being much larger than that of those who were “at high risk” (this was also more than 1) suggest that current psychiatric history is a very important antenatal risk factor even for those who are not at high risk of depression during pregnancy. However, the actual mental status of women without depression during pregnancy can be overlooked by perinatal professionals. We suggest that pregnant women with current psychiatric history, even if they were not depressive, should be carefully monitored for having the potential for high risk of PND.

### Strengths and limitations

This cohort study was performed in a heavily populated ward (about 900,000 people). The facilities involved in the study allowed a diverse sample, ranging from those who cannot afford perinatal care fees to those who can access expensive, specialized services. Thus, the results in this study can be regarded as strong evidence for perinatal mental health.

There are, however, several limitations in this study. First, we assessed mental health at T1 and T2 only by EPDS. No clinical diagnosis was made using structured or clinicians’ interviews. Thus, we cannot confirm the participants’ mental health with clinical diagnosis. Nonetheless, the sensitivity and specificity of the cut-off score compared to the clinical diagnosis of major depression using the Japanese version of the Schedule for Affective Disorders and Schizophrenia are reliable (75% and 93%, respectively) [17]. Second, this study may not have included a sample that is representative of the entire Japanese population. The Setagaya ward is a residential area in metropolitan Tokyo. Therefore, the participants’ socioeconomic statuses are relatively higher compared to other areas. In addition, many of them were from nuclear families. In Japan, there is a higher prevalence of nuclear families in the major cities compared to the rural regions, where extended families are often important resources for post-delivery care [2, 39]. Third, the questions on psychosocial factors in the questionnaires used in this study were not ones that have been validated (e.g. social supports, family cohesion, experience of child abuse). Fourth, depression during pregnancy and the postnatal period may be accompanied by underlying bipolar disorder. Previous studies reported that underlying bipolarIIdisorder exists in 13% of women with high levels of depressive symptoms in pregnancy [40] and 22% in the postnatal period [41]. This study examined postpartum depression using EPDS only. We did not examine the possibility of bipolar depression or mixed state in the women with depression. Fifth, we used a cut-off score of 9 for EDS for antenatal depression (AND) in this study. Although a cut-off score of 9 has been validated for postnatal depression in a Japanese women sample, there is no validated cut-off score for AND in Japanese women. Several studies used the cut-off score of 10 [42, 43], 12 [44, 45] and 13 [46–48] antenatally and postanatally. Matthey et al. [49] suggested that the validated cut-off score for PND should be 13 or more, and 15 or more for AND in English-speaking women. Considering that the cut-off score for PND is 9 in Japanese women, the cut-off score for AND in Japanese women is likely to be lower than that in English-speaking women. In addition, the purpose of this study was to investigate the risk factor for PND from a population approach viewpoint. We targeted women at high risk of PND and not those with severe depressive state. Thus, we thought it would be more appropriate to use the unvalidated cut-off score of 9 rather than a higher one. Nonetheless, we acknowledged that the use of an unvalidated score was one limitation of this study. Sixth, the attrition rate of this study is not negligible (about one-third of participants who enrolled in this study were not analyzed in the multivariate analyses). As such, the results may not be representative of the general population.

### Suggestions for clinical implications and further research

The risk factors of PND revealed in this study (i.e., mental health, primipara, current psychiatric illness treatment, current physical illness treatment, and family cohesion) should be paid attentions by professionals in maternal and child health. In addition, we suggest that EPDS should be used checked during pregnancy to predict postnatal mental health as well as assessing mental health during pregnancy periods. Interestingly, the U.S. Preventive Services Task Force recommends “screening adults for depression (including postnatal, but not pregnant women) when staff-assisted depression care supports are in place to assure accurate diagnosis, effective treatment, and follow-up” [50]. These mental health screenings should be performed where support systems for those who are at risk of mental health problems are available. Many health professionals have come to realize the necessity of integrating mental health screening into routine primary care for pregnant and postpartum women, as well as to follow up this screening with treatment or referral and with follow-up care [9]. In Japan, one of the main reasons why antenatal mental health screening is not common is because support network systems for women with mental health problems have not been developed enough [51]. We suggest that such networks should be established before preforming antenatal mental health screening. Since our results included Japanese culture-based psychosocial aspects, further cross-cultural study about family cohesion and perinatal mental health are needed. The results of multicollinearity tests suggest that these risk factors are demonstrated independent risk factors for PND. In addition, certain combinations of psychosocial factors may be risk factors for PND [52]. Further research on combination psychosocial factors as risk factors for PND is needed. A validation study for the cut-off score for AND in a Japanese sample should also be performed. Furthermore, it would be worthwhile to reassess the antenatal risk factors for PND using validated cut-off score for EDS and to compare the results with those achieved in this study.
